# Factors Impacting Advance Decision Making and Health Care Agent Appointment among Taiwanese Urban Residents after the Passage of Patient Right to Autonomy Act

**DOI:** 10.3390/healthcare11101478

**Published:** 2023-05-18

**Authors:** Yi-Ling Wu, Chun-Yi Yang, Tsai-Wen Lin, Pei-Han Shen, Zong-Dar Tsai, Ching-Nu Liu, Chia-Chen Hsu, Samuel Shih-Chih Wang, Sheng-Jean Huang

**Affiliations:** 1 Taipei City Hospital, Taipei 103212, Taiwan; a4279@tpech.gov.tw (Y.-L.W.); a1127@tpech.gov.tw (C.-Y.Y.); a5234@tpech.gov.tw (P.-H.S.); a1921@tpech.gov.tw (Z.-D.T.); a3590@tpech.gov.tw (C.-N.L.); z3893@tpech.gov.tw (C.-C.H.); 2 Department of Health and Welfare, Tian-Mu Campus, College of City Management, University of Taipei, Taipei 111036, Taiwan; 3 National Academy of Education Research, Taipei 237201, Taiwan; 898010029@ntnu.edu.tw; 4 National Taiwan University Hospital, Taipei 100225, Taiwan; sjhuang1@ntu.edu.tw

**Keywords:** advance care planning, advance directives, health care agent, Patient Right to Autonomy Act

## Abstract

In recent years, advance care planning (ACP) promotion in Taiwan has expanded beyond clinical practice to the broader population. This study aims to investigate people’s attitudes toward ACP and to identify factors influencing their signing of advance directives (ADs) and appointment of health care agents (HCAs). Methods: We identified 2337 ACP participants from consultation records between 2019 and 2020. The relationships among the participants’ characteristics, AD completion, and HCA appointment were investigated. Results: Of 2337 cases, 94.1% completed ADs and 87.8% were appointed HCAs. Welfare entitlement (OR = 0.47, *p* < 0.001), the place ACP progressed (OR = 0.08, *p* < 0.001), the participation of second-degree relatives (OR = 2.50, *p* < 0.001), and the intention of not being a family burden (OR = 1.65, *p* = 0.010) were significantly correlated with AD completion. The probability of appointing HCAs was higher in participants with family caregiving experience (OR = 1.42, *p* < 0.05), who were single (OR = 1.49, *p* < 0.05), and who expected a good death with dignity (OR = 1.65, *p* < 0.01). Conclusions: Our research shows that adopting ACP discussion in Taiwan is feasible, which encourages ACP conversation and facilitates AD completion. Implications: Male and younger adults may need extra encouragement to discuss ACP matters with their families. Limitations: due to sampling restrictions, our data were chosen from an urban district to ensure the integrity of the results. Furthermore, interview data could be collected in future research to supplement the quantitative results.

## 1. Introduction

In recent decades, developed countries have enacted legislation to protect patient autonomy and encourage patients to make advance medical plans, such as the Patient Self-Determination Act of the United States (2003), the Mental Capacity Act of the United Kingdom (2005), and the Patientenverfügungsgesetz of Austria (2006) and Germany (2009). These bills allow people to make decisions when they are healthy and discuss their preferences with their doctors and relatives, who subsequently carry out their decisions and preferences [1]. The Patient Right to Autonomy Act (PRAA), enacted on 18 December 2015 and put into effect on 6 January 2019, was the first law protecting patient autonomy in Asia. It allows people with full capacity to sign and register an advance directive/decision (AD) through a consultation procedure—advance care planning (ACP). The AD declarant can appoint a health care agent (HCA) via written consent, who will receive information and communicate the patient’s intentions if the declarant becomes incapacitated or unable to express their wishes adequately in the future. This written and signed statement describes a person’s willingness to accept or deny life-sustaining treatment (LST), artificial nutrition and hydration (ANH), or other types of medical care in order to ensure a good death under specific clinical conditions.

People from different demographic and cultural backgrounds may have different perspectives on patient autonomy [2]. Several studies have indicated that factors such as older age, female gender, higher education [3], being Caucasian [4], or being a white-collar worker influence AD completion. A person who is familiar with palliative care [5] or who has a positive attitude toward AD and respects other people’s decisions is more likely to complete AD [6]. One study found that native Taiwanese people with a higher quality of life and more knowledge about ACP are more likely to sign an AD [7]. Some researchers, however, indicate that there are no significant age and gender differences in those who complete ADs [8]. In terms of HCA designation, surrogate decision-making is frequently required for elderly Americans nearing the end of life, and both AD and HCA have a significant impact on decision-making outcomes [9]. Patients and the elderly who have family members usually choose their family members to be their HCAs [10]. On the other hand, declarants without relatives may appoint healthcare professionals, lawyers, or friends as their HCAs [11]. A systematic review found that HCAs or surrogates correctly conjectured 68% of patients’ treatment preferences [12].

Many Asian regions, including Japan, South Korea, Hong Kong, and Singapore, are heavily influenced by Confucianism, making it challenging to begin ACP discourse and limiting patient autonomy in end-of-life decisions [13,14,15,16]. Indeed, this may be a myth of filial piety or parental respect, because the AD process and the completion of ACP increase patient hope [17], improve end-of-life care and patient and family satisfaction, and reduce stress, anxiety, and depression in surviving relatives [18]. Previous studies of ACP in Asians only included the elderly, those nearing the end of their lives, or those in the hospital. In few studies, Asian non-terminal or non-elderly individuals have been surveyed about their experiences with ACP consultations in outpatient clinics, the completion of AD, and the appointment of an HCA. In fact, frail elders are expected to represent a significant share of palliative care users, despite not having been recognized as such until recently [19].

Death is a sensitive and challenging subject in Taiwan, and many patients do not communicate their preferences when they are still capable of making their own decisions. Before the passage of the PRAA, disease conditions were frequently hidden from end-of-life patients. It was common for families to make medical decisions with advice from doctors when the capacity of a patient was deficient [20]. Consequently, disagreements, confrontations, and legal issues often occurred among physicians, patients, and families [21]. ACP consultation can help patients, families, and medical teams communicate with one another [22], so that ADs can be completed before the patient’s physical and mental abilities deteriorate [23,24]. In Taiwan, the PRAA promotes anticipatory conversations regarding end-of-life treatment options when a terminal disease appears, and since then, 20,973 ADs have been completed. This study aimed to investigate people’s attitudes toward participating in ACP, and to identify factors influencing an individual’s decisions regarding AD and whether to designate an HCA, among adults attending ACP consultation, following the clinical practice of PRAA promotion to a broader range of the population. Hence, public awareness about ACP consultation and the signing of ADs in Taiwan can be improved. Further, our findings could encourage the signing of ADs in nations or cultures where death is a taboo topic.

## 2. Materials and Methods

This research was a retrospective study. An outpatient, inpatient, and in-home ACP consultation program was implemented by the Taipei City Hospital, Taiwan, in all branches. The program included ACP communication and AD signing, and noting on national health insurance identification cards. Individuals with legal ability who were at least 20 years old were eligible to participate in ACP consultations. This large city hospital in Taipei, with seven branches, provided ACP consultations to 2337 persons between 6 January 2019, and 5 January 2020. A total of 2198 of the participants completed an AD.

The study was approved by the institutional review board (TCHIRB-108XX008-E) of TXX. In this secondary data study, participants’ ADs and the records of their ACP consultations were used. Following the ACP conversation, the participants determined whether to appoint an HCA and reached an AD agreement on choices of LST and ANH. The records of the ACP consultation included the following information: gender, age, welfare entitlement, disease conditions, family caregiving experience, location of ACP consultation, whether second-degree relatives participated, and the reason for participating in ACP. The participants’ reasons for participating in an ACP consultation were also determined based on their responses to the counselor’s questions: “what are your reasons for making an AD? My reasons are (1) I am suffering from a disease; (2) I am single; (3) I hope for a good death with dignity; (4) I heard from media reports and propagations; (5) I consider planning for the end of my life; (6) My family member is suffering from a disease; (7) I am reluctant to let my family members taking responsibility for decision-making; (8) I avoid becoming a burden to my family members”. Although an open question allows greater possibilities and complexity, categorization of the provided answers would be an arduous task. The exact meanings could be difficult to determine due to the implicit nature of communication related to death. Thus, we defined the participants’ reasons for completing an AD.

Data were analyzed using SPSS 22.0 (IBM Corp, Armonk, NY, USA). All nominal and ordinal variables were described using frequencies. After verification, the raw data presented a normal distribution. We used the Chi-square test, Fisher’s exact test, independent samples *t*-test, bivariate logistic regression, and multivariate binary logistic regression with an Omnibus test, a Hosmer–Lemeshow goodness of fit test, and Cox–Snell and Nagelkerke tests to analyze the relationships between sociodemographic factors and whether Ads were completed and HNAs appointed.

## 3. Results

### 3.1. Characteristics of Study Participants

Table 1 summarizes the sociodemographic characteristics of the 2337 participants who had received an ACP consultation. The overall mean (SD) age was 60.45 (14.09), and more than 55.0% of the participants were over 60. Among them, 65.3% were female, and 34.7% were male. Approximately 30.0% of the participants had family caregiving experience, among which 71.0% were female, and 12.0% had welfare entitlement. A total of 53.4% of participants did not have self-reported diseases, 14.2% participants had self-reported diseases, and 32.4% did not respond. Of the 332 participants who had self-reported diseases, 129 (38.9%) had malignancy, 74 (22.3%) had a history of stroke or cardiovascular diseases, 69 (20.8%) had mental disorders, 49 (14.8%) had neurodegenerative diseases, and 45 (13.6%) had liver cirrhosis or any organ failure. Regarding the locations, 2233 ACP consultations took place in a hospital outpatient clinic, 49 in hospital admission, 21 at home, and 34 at the institution. In terms of participants’ reasons for completing an AD, 1548 participants (66.2%) reported expecting a good death with dignity, 1476 (63.2%) reported planning for the end of life, 1041 (44.5%) reported reluctance to let their family members take responsibility for decision-making, 921 (39.4%) reported not wanting to become a burden to their family members, 225 (9.6%) reported being single, 319 (13.6%) reported family members suffering from disease, 169 (7.2%) reported their own suffering from disease, and 152 (6.5%) reported being influenced by media reports and propagations.

Of the 2337 participants who had received ACP services, 2198 (94.1%) had completed an AD, and 139 (5.9%) had not. In the bivariate analysis (Table 2), participants who had not accomplished AD signing comprised a significantly higher proportion of those with welfare entitlement (21.6%), who had performed ACP at an institution (10.1%), and who did not have the participation of second-degree relatives (23.8%). Participants who accomplished AD signing comprised a significantly higher proportion of those with the intention of not being a family burden (40.2%). The proportion of reported neurodegenerative diseases was higher among participants who had not completed AD signing (8.2%) than in those who had completed AD signing (3.5%). The results of the univariate binary logistic regression in Table 3 reveal that welfare entitlement (OR = 0.47, *p* < 0.001), the location of ACP progression (OR = 0.08, *p* < 0.001), the participation of second-degree relatives (OR = 2.50, *p* < 0.001), and, compared to the group of participants who had not signed an AD, the intention of not being a family burden (OR = 1.65, *p* = 0.010) were factors that were significantly correlated with signing an AD.

For multivariate binary logistic regression, the proportion of uncompleted AD signing was associated with the place where participants conducted ACP in the institution (adjusted odds ratio (AOR) = 0.14, 95.0% CI 0.06–0.35) (Table 3). After considering the influences of other factors, the proportion of not accomplishing AD signing was highest in participants with welfare entitlement, without the participation of second-degree relatives, with the intention to not be a family burden, and with self-reported degenerative diseases, without statistical difference. The Omnibus model coefficient test reached the significance threshold (*p* < 0.001), indicating that at least one of the seven independent variables effectively explains and predicts the categorical results of the AD-completed condition. The Hosmer–Lemeshow goodness of fit test (*p* = 0.142) was not significant, with Cox–Snell R^2^ = 0.02 and Nagelkerke R^2^ = 0.062.

### 3.2. HCA Appointment

Among 2337 cases of ACP services, the proportion of participants who were not appointed HCAs was 87.8%, and that of participants who were appointed HCAs was 11.3%. In the bivariate analysis, significant differences were found between the participants who were appointed and who were not appointed HCAs in the variables of family caregiving experience, a self-reported neurodegenerative disorder, and specific consultation reasons, such as being single, expecting a good end with dignity, prior life arrangement, and family members suffering from disease (Table 4).

The presence of family caring experience (OR = 1.42, *p* < 0.05), being single (OR = 1.49, *p* < 0.05), and expecting a decent death with dignity (OR = 1.65, *p* < 0.01) were the variables significantly affecting the appointment of HCA in the univariate binary logistic regression. The final multivariable binary logistic regression showed that the probability of appointing an HCA was 1.47 times higher in participants with family caregiving experience than in persons without experience (AOR = 1.47, *p* < 0.05). Furthermore, the likelihood of appointing an HCA in participants who were single was 2.53 times higher than that in participants without a reason to engage in ACP (AOR = 2.53, *p* < 0.001). Finally, the probability of appointing an HCA was 1.45 times higher in participants expecting a good death with dignity than in participants with no expectations. The comparison was not statistically different when the effects from other explanatory variables were concerned (AOR = 1.448, *p* = 0.054). The omnibus model coefficient test reached the significance threshold (*p* = 0.002), indicating that among the seven independent variables, at least one could effectively explain and predict the categorical results of the samples in the condition where an HCA was not appointed. The Hosmer–Lemeshow goodness of fit test (*p* = 0.692) did not reach the significance threshold, with Cox–Snell R^2^ = 0.01 and Nagelkerke R^2^ = 0.03.

In the absence of HCA, the omnibus model coefficient test attained the significance threshold (*p* = 0.002), revealing that at least one of the seven independent variables could adequately explain and predict the categorical findings. Moreover, the Hosmer–Lemeshow goodness of fit test (*p* = 0.692) was not significant, with Cox–Snell R^2^ = 0.01 and Nagelkerke R^2^ = 0.03.

## 4. Discussion

Because national health insurance in Taiwan does not cover ACP consultations, all participants in this study had to pay for the service and set aside around 2 h. The setting aside of extra time implies that the participants might better understand palliative care and have a substantial commitment to good death. Over 53.0% of all participants revealed that they were in good health, with no life-threatening diseases or chronic problems. Our study discovered that women (65.3%) and the elderly (55.0%) were more willing to participate in ACP consultations about their future medical care, and there was no significant difference in gender or age strata among AD signing and HCA appointments. Previous studies also observed similar gender and age distributions of ACP participants. According to the national statistics in Taiwan, women accounted for 69.1% of those who registered their willingness to establish a DNR order. They received palliative care on their health insurance IC cards between 2006 and 2012, with 89.8% being over 40 and 50.0% being 50–69 years old [25]. An American study indicated that the rate of signing ADs in elderly persons could be as high as 60.0% [26], higher than the signing rates in the general population (1–30.0%) [27].

This study found that participants who had not completed AD signing comprised a higher proportion of those with welfare entitlement, who had performed ACP in an institution, and who did not have the participation of second-degree relatives. Although the Taipei City Hospital offered ACP consultation fee subsidies to low-income people, older adults living alone, and people with disabilities, these groups accounted for only a small percentage of the ACP participants. Several systematic review and meta-analysis studies focusing on Asian studies have yielded interesting syntheses on family factors. Family roles were important themes in Eastern societies [28]. The initiation of ACP was dominated by family factors and influenced by family beliefs [29]. Although the involvement of family members is important in the end-of-life process, the issue of ACP has possibly been avoided by most patients and their families in Chinese society. As demonstrated by Xu et al., who explored and compared AD and preferences for end-of-life care among older nursing home residents in Hong Kong and Taiwan, only 14% of Hong Kong participants and 18% of Taiwan participants reported prior occurrence of end-of-life care discussions with their family members or health professionals [30]. The role of the participation of second-degree relatives in completing AD is unclear; it might implicate the contribution of opinions from specific family members, especially the grandchildren whom the patient cares for or loves. This perspective remains to be solidarized and investigated. Many studies have found that deficiencies in educational attainment, information, and socioeconomic status, as well as specific race factors, significantly reduce AD completion. For example, ethnic minorities lacked access to medical and legal professionals and related official documents, faced literacy and language barriers, had a deep-rooted distrust of physicians and medical providers or facilities, and were unwilling to make health care decisions because they could not fully understand the documents [31]. Thus, ACP counselors should develop specific approaches using accessible and understandable documents to assist disadvantaged groups in comprehending the importance and benefits of ACP. It is a critical concern for the future promotion of ACP.

The success rate of AD signing was significantly lower among participants who were inhabitants of long-term care facilities in this study. The differences in participant characteristics could explain some features of the institutions where ACP was performed. Most participants who had performed ACP in an outpatient clinic were healthy (54.0%), with only 7.2% of consultations occurring due to the patients suffering from disease. Patients who had received ACP on admission or at home, on the contrary, were most likely severely or terminally ill. Previous studies reported that an influence of institutional culture on ACP, e.g., overly prescriptive and “check box” ways of carrying out ACP, hinders rather than promotes high-quality end-of-life treatment [32,33]. It is likely that institutionalized ACP procedures and documentation inadequately reflect end-of-life care interactions with patients. Some health professionals suggest a more personalized, informal approach to arranging treatment for their patients [32,34].

Although completing an AD is a matter of individual autonomy, family members frequently play a vital role in decision-making. In our study, 10.14% of participants underwent ACP without the presence of their relatives. Unless all relatives are deceased, missing, or have specific reasons for exemption, the PRAA requires at least one relative of first- or second-degree affinity to participate in the ACP consultation. This regulation alleviates the plight of people who live alone and without family. However, relatives who did not attend ACP for various reasons, such as being too busy to participate, or having conflicts or alienation in their relationships, are still very likely to be involved in medical decision-making in the future. Therefore, ACP medical teams need to remind the participants that they must proactively inform their relatives about their follow-up care plan to reduce future communication difficulties among family members when implementing medical decisions.

More than 94.0% of the participants in our study had completed an AD. Moreover, a significantly higher proportion of participants who had accomplished AD signing had the intention of not being a family burden. Providing care for senior family members has long been considered a societal obligation or duty to requite previous care received from them [35]. Although this thought may raise the desire to care, it could lead to distress and burden. In this context, the perception of being a burden to others influenced end-of-life care decisions in Japan [35] and the United States [36]. Furthermore, appointing an HCA is an optional and non-mandatory decision according to the PRAA. When participants are unconscious or unable to express their wishes clearly, the designated HCA must express medical wishes on their behalf. The responsibilities of an HCA include listening to medical instructions, signing examinations and treatment-related agreements, and carrying out pre-approved medical decisions based on the wishes of the declarant. In this study, 87.8% of the participants did not appoint an HCA, and only 11.3% of them appointed an HCA. Why was the proportion of HCA appointments so low? We observed some concerns and worries from the participants, which appear to be related to impracticality; for example, some believe it is a burden to the HCA to decide for others; some refer to the unpredictability of the future, for instance, the HCA may die sooner; and some are concerned about possible differences in opinions on medical preferences.

Our study found that being single, having family caregiving experience, and expecting a good death with dignity significantly influenced HCA appointment. One study reported that marital status strongly affects the variations of intention to AD completion. These findings indicated that married persons were less inclined to consider setting up an AD, since their spouses already understand their medical care preferences. The close and constant contact of spouses gives more opportunity for an informal discussion about medical care preferences. On the other hand, individuals who were not married were convinced that their family members or acquaintances are unaware of their health care choices and may seek formal advance directives to ensure that their medical care preferences are properly communicated [37].

Moreover, informal caregivers provide an essential service to their family members while dealing with pressure and stress, contributing to low subjective well-being [35]. This pressure and stress may cause caregivers to consider what they can do in the future to avoid burdening family members when they are ill and require assistance, leading them to feel compelled to discuss ACP or appoint an HCA to manage their future care [38]. Furthermore, patients who claimed to have discussed treatment choices with their surrogates and told them about their preferences showed higher levels of agreement [39]. Surrogates who have learned about their patient preferences are more aware of what is essential when approaching death [39,40]. Surrogates and physicians may be able to more appropriately satisfy patient preferences due to these conversations [40,41]. Patients and family members agreed on preferences that also included more intangible and emotional sentiments, such as maintaining dignity and self-respect, reducing worry and strain on loved ones, being at peace with death, being afraid of dying, and receiving hugs [39].

Previous studies have found that ACP has both formal and informal components [35,42]. Informal discussions with family members or health care providers are part of the ACP process. The goal of end-of-life conversations has moved from completing ADs to sharing discussion processes regarding ACP that involve patients, surrogates, and doctors [42]. ACP talks enhance the quality of end-of-life care while reducing emotional trauma in families [35]. In the absence of an AD, civil law in Taiwan indicates that the priority of medical decisions is based on the seniority ranking of the relatives. This causes chaos in some families. Typically, certain senior family members who seldom engage in the treatment may emerge when the patient nears the end of life. They reject or alter the choice of most of the family members. These family disputes hamper the provision of high-quality care [43]. Therefore, an early open dialogue of values and preferences among declarants, HCAs, relatives, and medical personnel can inform, empower, and reduce future conflicts in clinical decision-making. Investigation of the influences of family caregiving experience and being single on HCA appointments, and the possible mechanisms, is necessary for future practice. The phenomenon of participants with single status and family caregiving experience tending to appoint HCAs may warrant further assessment for the development of strategies in clinical practice.

## 5. Conclusions

Because Taiwan’s palliative care education has been vigorously promoted since the passage of the PRAA legislation, and Taipei City Hospital is a trial promotion unit, such a high completion rate is also evidence of promoting breakthroughs. Males and young people may require more encouragement to bring up ACP issues to their families. Medical care professionals can actively deliver information, convey respect for patient wishes and autonomy, and facilitate ACP for institutional and inpatient residents. The involvement of family members in implementing medical decisions will require more support in the future. We have identified factors that influence the desire to participate in ACP, and determined their implications for policy-making, practical practice, professional education, and academic research.

Our study found that the majority of participants requesting ACP consultations reported being in good health at the time of the survey, but also expressed a willingness to engage in ACP and make decisions about end-of-life care. This suggests that there may be a disconnect between individuals’ current health status and their desire to plan for future care needs. Patients and their families may be unable to make timely decisions that satisfy their care needs or expectations due to unforeseen circumstances or severe diseases, in addition to last-minute discussions regarding palliative care programs. Therefore, it is beneficial to proactively encourage communities to participate in ACP consultation. We recommend the implementation of community death education pathways to foster awareness among institutions and communities regarding the importance of reflecting on one’s finitude, thinking about one’s end-of-life choices, and fighting the taboo regarding death and its censorship [44]. Preferences and willingness may change as one’s health status changes. What the PRAA has provided regarding changes in ADs after signing is an essential topic that warrants further investigation.

## 6. Limitations and Future Research

Last but not least, due to sampling restrictions, our data were chosen from an urban district to ensure the integrity of the results. Furthermore, interview data could be collected in future research to supplement the quantitative results. Additionally, considering the impact of the COVID-19 pandemic, a comparison between the pre- and post-pandemic period could also be a research topic.

## Figures and Tables

**Table 1:** Demographic Characteristics of Participants.

Variables	*n* (2337)	%
Gender		
Male	812	34.7%
Female	1525	65.3%
Age		
Below 30 years	74	3.2%
31–40 years	168	7.2%
41–50 years	272	11.6%
51–60 years	559	23.9%
61–70 years	713	30.5%
71–80 years	394	16.9%
Above 80 years	157	6.7%
Family caregiving experience		
Without experience	1034	44.2%
With experience	688	29.4%
Caregiver’s gender		
Male	199	28.9%
Female	489	71.1%
Welfare identity		
General public (no welfare identity)	2056	88.0%
With welfare identity (all)	281	12.0%
Disease condition		
No self-reported diseases	1247	53.4%
With diseases (clinical conditions related—all)	332	14.2%
No response	758	32.4%
Disease conditions—disease types		
Cancers	129	38.9%
History of stroke or cardiovascular diseases	74	22.3%
Mental disorders	69	20.8%
Neurodegenerative disorders	49	14.8%
Liver cirrhosis or any organ failure	45	13.6%
AD Completion		
AD Uncompleted	139	5.9%
AD Completed	2198	94.1%
The place ACP progressed		
Hospital (outpatient clinic)	2233	95.5%
Hospital (admission)	49	2.1%
Home	21	0.9%
Institution	34	1.5%
Participation of second-degree relatives in ACP		
No	244	10.4%
Yes	1818	77.8%
Reasons for ACP consultation		
Own suffering from disease	169	7.2%
Being single	225	9.6%
Expecting a good death with dignity	1548	66.2%
Prior life arrangement	1476	63.2%
Media reports and propagations	152	6.5%
Family members suffering from disease	319	13.6%
Do not wish family members to take responsibility for making decisions	1041	44.5%
Do not want to be a family burden	921	39.4%

**Table 2:** Results of Bivariate Analysis and Univariate Binary Regression of Factors Associated with Advance Directive Completion or Lack of Completion.

	Bivariate Analysis						
Variables	AD Uncompleted (*n* = 139, 5.9%)			AD Completed (*n* = 2198, 94.1%)			*p*-Value
	*n*		%	*n*		%	
Gender							0.157 ^a^
Male	56		40.3%	756		34.4%	
Female	83		59.7%	1442		65.6%	
Age							
Mean	59.05	±	14.63	60.53	±	14.05	0.228 ^b^
Below 40 years	18		12.9%	224		10.2%	0.299 ^a^
41–65	71		51.1%	1050		47.8%	
Above 65 years	50		36.0%	924		42.0%	
Family caregiving experience							0.741 ^a^
Without experience	65		58.6%	969		60.1%	
With experience	46		41.4%	642		39.9%	
Caregiver’s gender							0.568 ^a^
Male	15		32.6%	184		28.7%	
Female	31		67.4%	458		71.3%	
Welfare entitlement							<0.001 ***^,a^
General public (no welfare entitlement)	109		78.4%	1947		88.6%	
With welfare entitlement	30		21.6%	251		11.4%	
Disease condition							0.379 ^a^
No self-reported diseases	67		75.3%	1180		79.2%	
With diseases (clinical conditions related—all)	22		24.7%	310		20.8%	
Disease conditions—disease types							
Cancers	7		9.5%	122		9.4%	0.980 ^a^
History of stroke or cardiovascular diseases	4		5.6%	70		5.6%	0.000 ^c^
Mental disorders	5		6.9%	64		5.1%	0.421 ^c^
Neurodegenerative disorders	6		8.2%	43		3.5%	0.053 ^c^
Liver cirrhosis or any organ failure	3		4.3%	42		3.4%	0.732 ^c^
The place ACP progressed							<0.001 ***^,c^
Hospital (outpatient clinic)	123		88.5%	2110		96.0%	
Hospital (admission)	1		0.7%	48		2.2%	
Home	1		0.7%	20		0.9%	
Institution	14		10.1%	20		0.9%	
Participation of second-degree relatives in ACP							<0.001 ***^,a^
No	29		23.8%	215		11.1%	
Yes	93		76.2%	1725		88.9%	
HCA appointment							0.082 ^a^
No	114		93.4%	1938		88.3%	
Yes	8		6.6%	257		11.7%	
Reasons for ACP consultation							
Own suffering from disease	15		10.9%	154		7.0%	0.091 ^a^
Being single	9		6.5%	216		9.9%	0.199 ^a^
Expecting a good death with dignity	87		63.0%	1461		66.7%	0.384 ^a^
Prior life arrangement	86		62.3%	1390		63.4%	0.796 ^a^
Media reports and propagations	4		2.9%	148		6.8%	0.075 ^a^
Family members suffering from disease	23		16.7%	296		13.5%	0.294 ^a^
Do not wish family members to take responsibility for making decisions	64		46.4%	977		44.6%	0.679 ^a^
Do not want to be a family burden	40		29.0%	881		40.2%	0.009 **^,a^

^a^ Chi-square test. ^b^ Independent samples *t*-test. ^c^ Fisher’s exact test. ** *p* < 0.01, *** *p* < 0.001.

**Table 3:** Results of Univariate Binary and Multivariate Binary Regression of Factors Associated with Advance Directive Completion or Lack of Completion.

	Univariate Binary Regression						Multivariate Binary Regression		
Variables	AD Uncompleted (*n* = 139, 5.9%)			AD Completed (ref: AD Uncompleted)					
	*n*		%	Crude	95%CI	*p*-Value	Adjusted OR	95%CI	*p*-Value
				OR					
Gender									
Male	56		40.30%	1			1	(0.70–1.57)	0.811
Female	83		59.70%	1.29	(0.91–1.83)	0.158	1.05	(1.00–1.03)	0.114
Age				1.01	(1.00–1.02)	0.228	1.01		
Mean	59.05	±	14.63						
Below 40 years	18		12.90%	1					
41–65	71		51.10%	1.19	(0.70–2.03)	0.529			
Above 65 years	50		36.00%	1.49	(0.85–2.60)	0.17			
Family caregiving experience									
Without experience	65		58.60%	1					
With experience	46		41.40%	0.94	(0.63–1.38)	0.74			
Caregiver’s gender									
Male	15		32.60%						
Female	31		67.40%						
Welfare entitlement									
General public	109		78.40%	1			1		
(no welfare									
entitlement)									
With welfare entitlement	30		21.60%	0.47	(0.31–0.72)	<0.001 ***	0.73	(0.42–1.27)	0.262
Disease condition									
No self-reported diseases	67		75.30%	1					
With diseases (clinical conditions related—all)	22		24.70%	0.8	(0.49–1.32)	0.38			
Disease conditions—disease types									
Cancers	7		9.50%						
History of stroke or cardiovascular diseases	4		5.60%						
Mental disorders	5		6.90%						
Neurodegenerative disorders	6		8.20%						
Liver cirrhosis or any organ failure	3		4.30%						
The place ACP progressed									
Hospital (outpatient clinic)	123		88.50%	1			1		
Hospital (admission)	1		0.70%	2.8	(0.38–20.44)	0.311	2.68	(0.36–19.91)	0.336
Home	1		0.70%	1.17	(0.16–8.76)	0.881	0.84	(0.11–6.47)	0.868
Institution	14		10.10%	0.08	(0.04–0.17)	<0.001 ***	0.14	(0.06–0.35)	<0.001 ***
Participation of second-degree relatives in ACP									
No	29		23.80%	1			1		
Yes	93		76.20%	2.5	(1.61–3.89)	<0.001 ***	1.68	(1.00–2.84)	0.05
HCA appointment									
No	114		93.40%	1					
Yes	8		6.60%	1.89	(0.91–3.92)	0.09			
Reasons for ACP consultation									
Own suffering disease from disease	15		10.90%	0.62	(0.35–1.09)	0.094			
Being single	9		6.50%	1.57	(0.79–3.12)	0.202			
Expecting a good death with dignity	87		63.00%	1.17	(0.82–1.67)	0.384			
Prior life arrangement	86		62.30%	1.05	(0.74–1.50)	0.796			
Media reports and propagations	4		2.90%	2.43	(0.89–6.65)	0.085			
Family members suffering from disease	23		16.70%	0.78	(0.49–1.24)	0.296			
Do not wish family members to take responsibility for making decisions	64		46.40%	0.93	(0.66–1.31)	0.679			
Do not want to be a family burden	40		29.00%	1.65	(1.13–2.40)	0.010 *	1.29	(0.86–1.94)	0.221

* *p* < 0.05, *** *p* < 0.001.

**Table 4:** Results of bivariate analysis, univariate binary regression, and multivariate binary regression of factors associated with health care agents being appointed or not being appointed.

	Bivariate Analysis							Univariate Binary Regression			Multivariate Binary Regression		
Variables	HCA Unappointed (*n* = 139, 5.9%)			HCA Appointed (*n* = 2198, 94.1%)			*p*-Value	HCA Appointed (ref: HCA Unappointed)			HCA Appointed (ref: HCA Unappointed)		
								Crude OR	95%CI	*p*-Value	Adjusted OR	95%CI	*p*-Value
Gender													
Male	707		34.5%	98		37.0%		1					
Female	1345		65.5%	167		63.0%		0.90	(0.69–1.17)	0.416	1		0.112
Age								1.00	(0.99–1.01)	0.451	0.76		0.242
Mean	60.4	±	14.05	61.09	±	14.20	0.451 ^b^				1.01	(0.53–1.07)	
Below 40 years	211		10.3%	28		10.6%	0.868 ^a^					(1.00–1.02)	
41–65	988		48.1%	123		46.4%							
Above 65 years	853		41.6%	114		43.0%							
Family caregiving experience							0.034 *^,a^						
Without experience	951		60.8%	83		52.2%		1					
With experience	612		39.2%	76		47.8%		1.42	(1.03–1.97)	0.035 *	1		0.025 *
Caregiver’s gender							0.424 ^a^				1.47		
Male	180		29.4%	19		25.0%						(1.00–2.04)	
Female	432		70.6%	57		75.0%							
Welfare entitlement							0.108 ^a^						
General public (no welfare entitlement)			87.5%	241		90.9%		1					
With welfare entitlement	256		12.5%	24		9.1%		0.70	(0.45–1.08)	0.110	1		0.975
Disease condition							0.068 ^a^				0.99		
No self-reported diseases	1144		79.6%	103		73.0%						(0.60–1.64)	
With diseases (clinical conditions related—all)	293		20.4%	38		27.0%							
Disease conditions—disease types													
Cancers	115		9.1%	14		12.0%	0.315 ^a^						
History of stroke or cardiovascular diseases	68		5.6%	6		5.5%	0.963 ^a^						
Mental disorders	63		5.2%	6		5.5%	0.898 ^a^						
Neurodegenerative disorders	39		3.3%	9		8.0%	0.030 ^c^						
Liver cirrhosis or any organ failure	40		3.4%	5		4.6%	0.418 ^c^						
The place ACP progressed							0.339 ^c^						
Hospital (outpatient clinic)			95.2%	259		97.7%		1					
Hospital (admission)	45		2.2%	4		1.5%		0.46 ^d^	(0.20–1.06)	0.070	1		0.110
Home	20		1.0%	1		0.4%					0.42		
Institution	33		1.6%	1		0.4%						(0.15–1.215)	
Participation of second-degree relatives in ACP							0.489 ^a^						
No			12.0%	21		10.3%		1					
Yes	1636		88.0%	182		89.7%		1.18	(0.74–1.90)	0.490	1		0.214
Reasons for ACP consultation											1.43		
Own suffering from disease	140		6.8%	26		9.8%	0.075 ^a^					(0.81–2.517)	
Being single	190		9.3%	35		13.3%	0.041 *^,a^	1.49	(1.02–2.20)	0.042 *			<0.001 ***
Expecting a good death with dignity	1338		65.4%	200		75.8%	0.001 **^,a^	1.65	(1.23–2.22)	0.001 **	2.53		0.054
Prior life arrangement	1274		62.3%	192		72.7%	0.001 **^,a^				1.45	(1.53–4.17)	
Media reports and propagations	140		6.8%	11		4.2%	0.098 ^a^					(0.99–2.11)	
Family members suffering from disease	264		12.9%	48		18.2%	0.018 *^,a^						
Do not wish family members to take responsibility for making decisions	927		45.3%	104		39.4%	0.069 ^a^						
Do not want to be a family burden	823		40.2%	95		36.0%	0.185 ^a^						

^a^ Chi-square test. ^b^ Independent samples *t*-test. ^c^ Fisher’s exact test. ACP * *p* < 0.05. ** *p* < 0.01. *** *p* < 0.001. ^d^ The samples of ACP on admission, at home, and at the institution were categorized as one group, for they were likely to be terminally ill patients compared to those in the outpatient clinic.

## Data Availability

Data is unavailable due to privacy.

## References

[1] Wolf S.M., Boyle P., Callahan D., Fins J.J., Jennings B., Nelson J.L., Barondess J.A., Brock D.W., Dresser R., Emanuel L. (1991). Sources of concern about the Patient Self-Determination Act. N. Engl. J. Med..

[2] Ruhnke G.W., Wilson S.R., Akamatsu T., Kinoue T., Takashima Y., Goldstein M.K., Koenig B.A., Hornberger J.C., Raffin T.A. (2000). Ethical decision making and patient autonomy: A comparison of physicians and patients in Japan and the United States. Chest.

[3] Douglas R., Brown H.N. (2002). Patients’ attitudes toward advance directives. J. Nurs. Sch..

[4] Teno J.M., Gruneir A., Schwartz Z., Nanda A., Wetle T. (2007). Association between advance directives and quality of end-of-life care: A national study. J. Am. Geriatr. Soc..

[5] Lin H., Yang C., Chen M., Chiu T.Y., Hu W.Y. (2011). Inpatients’ willingness on and acceptance of promotion for signing of advance directives. TJHPC.

[6] Chan C.W.H., Wong M.M.H., Choi K.C., Chan H.Y.L., Chow A.Y.-M., Lo R.S.K., Sham M.M.K. (2019). Prevalence, perception, and predictors of advance directives among Hong Kong Chinese: A population-based survey. Int. J. Environ. Res. Public Health.

[7] Chang H., Yen C., Lin P. (2011). Perspectives on advance directives in outpatients of department of family medicine in Changhua City. TJHPC.

[8] Cugliari A.M., Miller T., Sobal J. (1995). Factors promoting completion of advance directives in the hospital. Arch. Intern. Med..

[9] Silveira M.J., Kim S.Y., Langa K.M. (2010). Advance directives and outcomes of surrogate decision making before death. N. Engl. J. Med..

[10] Coppolino M., Ackerson L. (2001). Do surrogate decision makers provide accurate consent for intensive care research?. Chest.

[11] High D.M. (1990). Who will make health care decisions for me when I can’t?. J. Aging Health.

[12] Shalowitz D.I., Garrett-Mayer E., Wendler D. (2006). The accuracy of surrogate decision makers: A systematic review. Arch. Intern. Med..

[13] Cheng S.-Y., Lin C.-P., Chan H.Y.-L., Martina D., Mori M., Kim S.H., Ng R. (2020). Advance care planning in Asian culture. Jpn. J. Clin. Oncol..

[14] Ng Q.X., Kuah T.Z., Loo G.J., Ho W.H., Wanger N.L., Sng J.G., Yang G.M., Tai B.C. (2017). Awareness and attitudes of community-dwelling individuals in Singapore towards participating in advance care planning. Ann. Acad. Med. Singap..

[15] Shin D.W., Lee J.E., Cho B., Yoo S.H., Kim S.Y., Yoo J.H. (2016). End-of-life communication in Korean older adults: With focus on advance care planning and advance directives. Geriatr. Gerontol. Int..

[16] Chung R.Y., Wong E.L., Kiang N., Chau P.Y., Lau J.Y.C., Wong S.Y., Yeoh E.-K., Woo J.W. (2017). Knowledge, attitudes, and preferences of advance decisions, end-of-life Care, and place of care and death in Hong Kong. A population-based telephone survey of 1067 adults. J. Am. Med. Dir. Assoc..

[17] Cohen M.G., Althouse A.D., Arnold R.M., Bulls H.W., White D.B., Chu E., Rosenzweig M.Q., Smith K.J., Schenker Y. (2022). Hope and advance care planning in advanced cancer: Is. there a relationship?. Cancer.

[18] Detering K.M., Hancock A.D., Reade M.C., Silvester W. (2010). The impact of advance care planning on end-of-life care in elderly patients: Randomised controlled trial. BMJ.

[19] Combes S., Nicholson C.J., Gillett K., Norton C. (2019). Implementing advance care planning with community-dwelling frail elders requires a system-wide approach: An integrative review applying a behaviour change model. Palliat. Med..

[20] Sun W.-N., Hsu H.-T., Ko N.-Y., Huang Y.T. (2020). Decision-making processes in surrogates of cancer patients in a Taiwan intensive care unit. Int. J. Environ. Res. Public Health.

[21] Huang H.-L., Tsai J.-S., Yao C.-A., Cheng S.Y., Hu W.Y., Chiu T.Y. (2020). Shared decision making with oncologists and palliative care specialists effectively increases the documentation of the preferences for do not resuscitate and artificial nutrition and hydration in patients with advanced cancer: A model testing study. BMC Palliat. Care.

[22] Cheung K.-C., Lau V.W.-K., Un K.-C., Wong M.S., Chan K.Y. (2017). Advance care planning for patients with advanced neurology diseases. Ann. Palliat. Med..

[23] Hickman S.E., Hammes B.J., Moss A.H., Tolle S.W. (2005). Hope for the future: Achieving the original intent of advance directives. Hastings Cent. Rep..

[24] Morrison R.S., Chichin E., Carter J., Burack O., Lantz M., Meier D.E. (2005). The effect of a social work intervention to enhance advance care planning documentation in the nursing home. J. Am. Geriatr. Soc..

[25] Li I.-F., Ching C.-H., Chuang R.-B., Du Y.Y., Chen H.W., Fang C.K. (2013). Effectiveness of hospice palliative care will recording on National Health Insurance IC card in Taiwan: 2006–2012. TJHPC.

[26] Kahana B., Dan A., Kahana E., Kercher K. (2004). The personal and social context of planning for end-of-life care. J. Am. Geriatr. Soc..

[27] Ho H.-L., Lin Y.-S., Wu Y.-C. (2015). Family members’ behavioral presentation during participation in terminal patients’ advance care planning. J. Ment. Health.

[28] Chikada A., Takenouchi S., Nin K., Mori M. (2021). Definition and Recommended Cultural Considerations for Advance Care Planning in Japan: A Systematic Review. Asia Pac. J. Oncol. Nurs..

[29] Martina D., Geerse O.P., Lin C.P., Kristanti M.S., Bramer W.M., Mori M., Korfage I.J., van der Heide A., Rietjens J.A., van der Rijt C.C. (2021). Asian patients’ perspectives on advance care planning: A mixed-method systematic review and conceptual framework. Palliat. Med..

[30] Xu X., Tu S.W., Lin C.C. (2021). Advance care planning preferences in Chinese nursing home residents: Results from two cross-sectional studies in Hong Kong and Taiwan. BMC Palliat. Care.

[31] Carr D. (2012). Racial and ethnic differences in advance care planning: Identifying subgroup patterns and obstacles. J. Aging Health.

[32] Johnson S., Butow P., Kerridge I., Tattersall M. (2016). Advance care planning for cancer patients: A systematic review of perceptions and experiences of patients, families, and healthcare providers. Psycho-Oncology.

[33] Boyd K., Mason B., Kendall M., Barclay S., Chinn D., Thomas K., Sheikh A., Murray S.A. (2010). Advance care planning for cancer patients in primary care: A feasibility study. Br. J. Gen. Pract..

[34] Cox K., Moghaddam N., Almack K., Pollock K., Seymour K. (2011). Is it recorded in the notes? Documentation of end-of-life care and preferred place to die discussions in the final weeks of life. BMC Palliat. Care.

[35] Miyashita J., Shimizu S., Azuma T., Takeshima T., Suzuki R., Fukuhara S., Yamamoto Y. (2021). Experience as an Informal Caregiver and Discussions Regarding Advance Care Planning in Japan. J. Pain Symptom Manag..

[36] Young A.J., Ofori-Boateng T., Rodriguez K.L., Plowman J.L. (2003). Meaning and agency in discussing end-of-life care: A study of elderly veterans’ values and interpretations. Qual. Health Res..

[37] Hopp F.P. (2000). Preferences for Surrogate Decision Makers, Informal Communication, and Advance Directives Among Community-Dwelling Elders: Results From a National Study. Gerontologist.

[38] Carr D. (2012). “I don’t want to die like that…”: The impact of significant others’ death quality on advance care planning. Gerontologist.

[39] Engelberg R.A., Patrick D.L., Curtis J.R. (2005). Correspondence Between Patients’ Preferences and Surrogates’ Understandings for Dying and Death. J. Pain Symptom Manag..

[40] Burns K.E., Prats C.J., Maione M., Lanceta M., Zubrinich C., Jeffs L., Smith O.M. (2017). The experience of surrogate decision makers on being approached for consent for patient participation in research. A multicenter study. Ann. Am. Thorac. Soc..

[41] Bryant J., Skolarus L.E., Smith B., Adelman E.E., Meuer W. (2013). The accuracy of surrogate decision makers: Informed consent in hypothetical acute stroke scenarios. BMC Emerg. Med..

[42] Carr D., Moorman S.M., Boerner K. (2013). End-of-life planning in a family context: Does relationship quality affect whether (and with whom) older adults plan?. J. Gerontol. B Psychol. Sci. Soc. Sci..

[43] Kramer B.J., Yonker J.A. (2011). Perceived success in addressing end-of-life care needs of low-income elders and their families: What has family conflict got to do with it?. J. Pain Symptom Manag..

[44] Ronconi L., Biancalani G., Medesi G.A., Orkibi H., Testoni I. (2023). Death Education for Palliative Psychology: The Impact of a Death Education Course for Italian University Students. Behav. Sci..

